# Chronic Myeloid Leukemia Patient’s Voice About the Experience of Treatment-Free Remission Failure: Results From the Italian Sub-Study of ENESTPath Exploring the Emotional Experience of Patients During Different Phases of a Clinical Trial

**DOI:** 10.3389/fpsyg.2019.00329

**Published:** 2019-02-20

**Authors:** Lidia Borghi, Sara Galimberti, Claudia Baratè, Massimiliano Bonifacio, Enrico Capochiani, Antonio Cuneo, Franca Falzetti, Alessandra Iurlo, Francesca Lunghi, Claudia Minotto, Ester Maria Orlandi, Giovanna Rege-Cambrin, Simona Sica, Sharon Supekar, Jens Haenig, Elena Vegni

**Affiliations:** ^1^Department of Health Sciences, San Paolo Hospital, University of Milan, Milan, Italy; ^2^Department of Clinical and Experimental Medicine, Section of Hematology, University of Pisa, Pisa, Italy; ^3^Department of Medicine, Section of Hematology, University of Verona, Verona, Italy; ^4^Department of Oncology, Azienda Toscana Nord Ovest, Livorno, Italy; ^5^Institute of Hematology, University of Ferrara, Ferrara, Italy; ^6^Institute of Hematology, Centro di Ricerca Emato-Oncologico (CREO), University of Perugia, Perugia, Italy; ^7^Hematology Division, Foundation IRCCS Ca’ Granda Ospedale Maggiore Policlinico, Milan, Italy; ^8^Hematology and Bone Marrow Transplantation Unit, San Raffaele Scientific Institute, Milan, Italy; ^9^UOS Hematology, UOC Oncologiaed Ematologia Oncologica AULSS3, Mestre, Italy; ^10^Hematology Unit, Fondazione IRCCS Policlinico San Matteo, Pavia, Italy; ^11^Division of Hematology and Internal Medicine, “San Luigi Gonzaga” University Hospital, Orbassano, University of Turin, Turin, Italy; ^12^Fondazione Policlinico Universitario Agostino Gemelli – IRCSS, Università Cattolica del Sacro Cuore, Rome, Italy; ^13^Oncology Region Europe, Novartis Farma SpA, Origgio, Italy

**Keywords:** chronic myeloid leukemia, nilotinib, ENESTPath, TFR, inner emotional experience, psycho-emotional outcomes

## Abstract

**Background:** The main objective of this study is to gain further insights on how chronic myeloid leukemia (CML) patients involved in an interventional clinical trial with the purpose of reaching treatment free remission (TFR) phase, perceived and experienced TFR failure. TFR failure was defined for the individual patient as either not being eligible for drug discontinuation or as having relapse in the TFR phase with reintroduction of nilotinib treatment.

**Methods:** Using a qualitative approach, out of 25 patients with CML who experienced TFR failure 14 were interviewed. Patients’ views and experiences were explored using in-depth interviews, analyzed using the Interpretative Phenomenological Analysis (IPA).

**Results:** The analysis of the interviews revealed that the experience of the diagnosis seems to have been lived as a traumatic break that has created a dichotomy, like an ambivalence in the ways in which CML patients perceived and experienced the whole disease journey, with contradictory feelings of both positive and negative emotions (e.g., a diagnosis of cancer, that is something distressing and of being afraid of, but also with a treatment and a life expectancies of which being grateful). This ambivalence of feelings was found to give meaning to the way in which patients cognitively and emotionally experienced the different steps of their disease history. Thus, four main issues, corresponding to different steps of the patients’ journey, were identified: (1) the moment of the diagnosis, (2) the experience of the illness journey: disease and treatment, (3) the moment of “TFR failure,” and (4) the impact of disease, treatment and relapse on the patient’s life.

**Conclusion:** This qualitative analysis helps in understanding patients’ perspective, both in terms of getting access to the inner subjective experience of having CML and its strict relationship with the involvement in a trial or its cessation. Clinicians should consider that the way in which CML patients feel engaged in a clinical trial, create expectancies about TFR or experience the TFR failure is linked to the process of coping with the diagnosis, which is characterized by ambivalence.

## Introduction

Chronic myeloid leukemia (CML) is a myeloid neoplasia characterized by the mutual translocation between chromosome 9 and 22 (Philadelphia chromosome) and by the BCR-ABL1 rearrangement, with a consequent synthesis of an uncontrolled tyrosine-kinase that induces increased proliferation, reduces apoptosis, and causes the entry of myeloid immature cells into the bloodstream (Rowley, 1973).

In the last decades, thanks to the introduction of first-generation tyrosine kinase inhibitors (TKIs) (imatinib), the molecular-targeted therapy in CML became a reality, with the consequent improvement of the outcome for more than 90% of cases. Notwithstanding imatinib already greatly improved overall survival and allowed patients to achieve high clinical response rates (Hanfstein et al., 2012), the introduction of second-generation TKIs (nilotinib and dasatinib) as first-line therapy allowed a higher percentage of patients to achieve deeper and faster molecular responses (MR). The excellent treatment option provided by TKIs are offering a survival comparable to that of the general population (Bower et al., 2016).

The newest guidelines of the National Comprehensive Cancer Network [NCCN] (2018) and of the European Society For Medical Oncology (ESMO) (Hochhaus et al., 2017) have recently proposed criteria for potential treatment discontinuation. In fact, in the latest years many studies clearly showed that about half of patients who achieved a sustained and deep molecular response (DMR) can discontinue treatment without losing their DMR, thus making the treatment-free remission (TFR) a reality. As recently published data suggest that the duration of DMR (Saussele et al., 2017) is important for maintaining TFR, the data derived from the ENESTPath trial will provide further insights. The primary objective of this trial is to elucidate the optimal duration of consolidation treatment before TFR attempt. Several variables have been identified as fundamental presupposition for TFR (length of therapy, depth of MR, type of TKI, line of treatment). Available data is mostly derived from interventional clinical trials with only Nilotinib having clear guidance for the TFR eligibility and the discontinuation procedure in the officially, health authority approved Summary of Products Characteristics, thereby leaving the process of decision making about interruption to each physician-patient dyad.

It is not difficult to imagine the emotional distress that this decision could induce, where patients could live this proposal as an unexpected and positive option, while others might see this moment as uncertain or risky. While patient’s view in the management of treatment has been well recognized even by the authorities that included patient reported outcomes (PRO) in the process of the new drug submission packages and regulatory approvals (Amir et al., 2012), there is still a lack of knowledge about the factors affecting emotional well-being of participants to clinical trials (Hopwood and Thatcher, 1990; Aaronson, 1992; Kaasa, 1992; Cox, 2003; Wineman et al., 2003). Most of the published studies use quantitative instruments to mainly assess quality of life (QoL), with only few stressing the importance of a qualitative methodology to enhance the comprehension of patients’ inner experience. A study published in 2003 (Cox, 2003) analyzed the emotional experiences of patients enrolled in phase I/II oncological trials. While the data obtained from the questionnaires revealed no changes over time, in-depth interviews adequately sketched some otherwise hidden aspects of the psychological, emotional, and social impact deriving from participation in a clinical trial from the perspective of the patient (Cox, 2003).

Focusing on CML, the majority of the already published studies assessed QoL using quantitative methods, such as the EORTC QLQ-CML24 (Efficace et al., 2012, 2014), FACT-LEU (Trask et al., 2013), or SF-36 (Ware and Sherbourne, 1992) questionnaire, producing some interesting results. In particular, these studies pointed out a lack of knowledge about TFR, as its emotional perception is yet to be adequately evaluated: in a series of 87 patients, 81% of them indicated that they would be willing to attempt TFR, especially those who experienced previous toxicities. On the other hand, the reluctance to discontinue treatment was often associated with a need for additional information and perhaps with a not fully confident relationship with the respective referent physician (Villemagne Sanchez et al., 2018).

In this panorama, we conducted a multilevel study employing a quantitative and qualitative approach for exploring CML patients’ psycho-emotional outcomes, QoL and inner experiences involved in different moments of disease history. In particular, the aim of this contribution is to present results from qualitative interviews about the subjective emotional experience of those patients who experience the TFR failure.

## Materials and Methods

### Study Design

The present contribution is part of an Italian sub-study examining patients’ psychological and emotional characteristics, involved in ENESTPath trial, a phase-3 European multicenter clinical trial involving 620 patients from 22 different countries. Enrolled patients received 12 months of nilotinib 300 mg BID as induction phase followed by further 12 months of consolidation. Patients achieving a sustained DMR (≥MR4 in 4 of 5 PCR assessments, including the last assessment, in the last 12 months) at the end of the first 24 months of treatment were either randomized to a further consolidation period of 12 months (Arm 2, 36 months of total treatment time) or discontinued immediately for TFR (24 months of total treatment). The primary endpoint of the ENESTPath is the assessment of the potential impact of a longer duration of consolidation (12 months vs. 24 months) on molecular relapse rate in the first 12 months of TFR (>MMR). All patients not in a stable MR4 and therefore not eligible for randomization according to the above mentioned criteria had to discontinue the trial.

The Italian sub-study involved 31 patients and assessed patients’ emotional experience during and after stopping nilotinib, and after trail discontinuation. To obtain a comprehensive evaluation of patients’ psycho-emotional outcomes and adopting a multilevel perspective, both quantitative (questionnaires) and qualitative (expressive writing and interviews) methods have been used.

This paper presents qualitative data gained from the interviews on patients who “failed” the trial, i.e., patients who had to start retreatment with Nilotinib because they relapsed in the TFR phase or patients who had to discontinue the trial because they were not eligible for randomization by month 24 after the induction and consolidation phase.

### Participants

Participants are all patients enrolled in the Italian sub-study who accepted to be interviewed because they experience the psychological phenomenon of “TFR failure” as they were ineligible for the randomization (unstable MR4 during the consolidation, early discontinuation of the main study: End of Phase – EOP) or they relapsed during TFR (loss of MMR, or the confirmed loss of MR4). Patient recruitment relied on convenience sample, as the group of interest was limited, as only Italian patients were involved. Participants were enrolled according to the maximum variability criterion, unless data saturation of the following variables: patients’ gender, patients’ age, years since CML-diagnosis.

The study was carried out in accordance with the GCP rules. As the Italian substudy is multicentric and involved many Centers (hospitals) all around Italy (Bari, Bologna, Cona, Livorno, Milano 2 Hospitals, Mirano, Orbassano, Pagani, Parma, Pavia, Perugia, Pescara, Pisa, Reggio Emilia, Roma 2 Hospitals, Taranto, Verona, Vicenza), the research protocol was approved by the Health Authorities, by the Ethics Commitee of the coordinator site (AO Università di Bologna Policlinico S. Orsola – Malpighi, Bologna, Italy) and by all the Ethics Commitee of all the involved Hospitals. All subjects gave written informed consent in accordance with the Declaration of Helsinki.

### Data Collection

Adopting a qualitative–interpretative methodology, in-depth unstructured interviews were conducted within one month from the trial discontinuation to explore patients’ emotional experience of the phenomena “being ineligible for randomization” or “failing TFR.” In-depth interviews have been widely used in the medical literature (Greenhalgh and Hurwitz, 1999), in particular when the aim is to reconstruct the meanings patients given to their experiences. Narration approach allows the speaker to reconstruct and reinterpret past, present and future events, making them understandable and communicable. Interviews were guided using open-ended questions (e.g., “Tell us about your illness experience, including the last trial period, starting from the point that is more important for you”) and followed patients’ narrative flow; the interviewer only facilitated the narration. Patients were asked to describe the course of their disease and their involvement in the clinical trial, including the final moments of trial discontinuation. Interviews were audio recorded and fully transcribed verbatim.

### Data Analysis

Verbatim reports were analyzed according to the principles of Interpretative Phenomenological Analysis (IPA) (Smith, 1996; Eatough and Smith, 2008; Smith et al., 2009), an inductive method of analysis that through an in-depth interpretation of individual narratives allows grasping the essence of how people attribute sense to their experiences (Conroy, 2003; Richards and Morse, 2007). IPA is based on the hermeneutic-phenomenology approach, in which a double hermeneutic focus is applied: the analyst interprets the participants’ interpretation of their lived experience. IPA is a widely used approach to qualitative research in health psychology, as it is particularly suited to discovering subjective processes through which patients make sense of their symptoms or diseases (see, for example, Griffiths et al., 2011; Hilgart et al., 2013; Vegni et al., 2013). For this reason, a level of homogeneity of experience (in this specific case, CML patients experiencing the discontinuation or return to treatment) is important in order to ensure access to a comparable experience across the group as a whole. Through the process of IPA, it is possible to uncover implicit beliefs and emotions that are carried in the stories by the deep analysis of their verbal statements, although writers are not necessarily completely aware of these psychological states from a cognitive viewpoint (Smith and Osborn, 2007). The analysis proceeds as a spiral from details to the whole, from one theme to the complexities of theme, from narratives to concepts, and again to narratives. Indeed, IPA is an iterative inductive process that starts from the detailed reading of the texts aimed at gaining a holistic understanding of all the collected narratives (Eatough and Smith, 2008).

In the present study, complete verbatim reports of the interviews were independently analyzed by two researchers (LB and EV), one of whom conducted the interviews. In the early stage of the analysis, the researchers took notes of their understanding of the text and of their preliminary insights about the meanings that participants attribute to their experience. A detailed case-by-case analysis of individual transcripts was conducted with the aim of identifying perceptions and emotions of patient’s journey through CML and the tria. Adeeper analysis was then conducted in order to further understand patient’s experience. Core themes were generated in two ways: (1) by clustering similar themes of the various accounts into more exhaustive categories, and (2) by formulating an interpretative hypothesis based on issues whose sense could not be understood separated but were instead interpreted globally as a whole. Preliminary results were discussed in depth adopting a triangular approach (a third researcher -SG- was involved), to reach an agreement on the themes. The analysis process was conducted during the final stage of data collection in order to verify the hypotheses that emerged from the interpretation process with patients. Finally, excerpts of the accounts were selected to exemplify the results.

## Results

The psychological Italian sub-study of ENESTPath enrolled 31 patients, of whom 25 patients experienced TFR failure; out of these 14 were interviewed as far as the phenomenon of TFR failure, including 8 ineligible for randomization due to unstable molecular response, and 6 patients who relapsed during TFR and had to be retreated. Patients were 11 males and 3 females, with a median age of 57.5 (range 36–82), and a relatively long history of disease (median = 10 years, range 3–15). Patients were mainly married (85.7%), with a medium-high educational level (58.3% high school, 25% graduates). The mean length of the interviews was 39′53″, ranging from 28′07″ to 53′54″.

The analysis of the interviews revealed an underlying theme that runs across the whole disease history, from the moment of the diagnosis to the experience of “TFR failure” due to unstable MR4: a dichotomy, like a split that seems to originate from a sort of traumatic break experienced by patients when they faced the CML diagnosis. This ambivalence of contradictory feelings with both positive and negative emotions (e.g., a diagnosis of cancer, that is something distressing and of being afraid of, but also with a treatment and a life expectancies of which being grateful) characterized the way in which CML patients cognitively and emotionally perceived and experienced the different steps of their disease journey.

Thus, four main issues, corresponding to different steps of the patients’ journey, were identified: (1) the moment of the diagnosis, (2) the experience of the illness journey: disease and treatment, (3) the moment of “TFR failure,” and (4) the impact of disease, treatment and relapse on the patient’s life.

### Experience of the Diagnosis

The first interesting observation is that all interviewed subjects, when asked to talk about the early “TFR failure,” started their narration from the experience of the diagnosis. Indeed, the majority of patients remembered the diagnosis itself in a dichotomous way: as a traumatic experience (in which the weight was greater when the patient’s perspective was far from that hypothesis), but at the same time as something wrong that was rapidly overcome, as “all goes well.”

“The doctor called me and told me it was leukemia... that moment was a blow, because I was really far from that hypothesis” [5].

“Fifteen years ago, when they told me I had this disease, I took it kind of hard at first, because I started to think about why this disease came huh... and I was worried I would no longer have that tranquility I had before” [6].

Interestingly, the words related to the diagnosis were not emotionally charged, but rather they appeared in a metaphoric and evocative way.

“I took two days to do all the investigations... it was just the day when the Twin Towers tragedy happened, I just saw it, I was hospitalized... in fact a parallelism.” [4]

Moreover, it was a seemingly common aspect that the moment of the diagnosis was not remembered in its details; nobody fixed the narration on the way or the sentences that the physician used for communicating the CML presence, as if that moment still represented a trauma from which patients tried to defend themselves from; however, patients still remembered doctors’ reassurances.

“The doctor told me: ‘yes you have leukemia... but... not all patients have the same leukemia, you have this type (CML), the others have the worst, you the best... you have to fight for your life …”’ [9].

“In the second visit after the exams the doctor wanted to explain to me... he reassured me, he said ‘no…’, he explained me that there’s like a crazed chromosome called Philadelphia that creates these... overhangs.. and he told me... ‘we have to keep it under control’... he just diagnosed me with chronic myeloid leukemia and told me it was not at acute levels, that it was not, well... serious, it was only to be taken care of, to be kept under observation and to be followed by a therapy” [11].

“The first moment I huh...I did not care so much, maybe because huh...the physician said to me ‘don’t worry, it’s not (serious), but you must do the blood tests’ ... later...when they told me the effective diagnosis hum... huh... I went home and I started crying” [11].

Finally, regarding the strategies used in coping with the diagnosis, patients reported to face the disease as something unexplainable and so difficult to control, lived in a passive way but precisely for these reasons they faced the diagnosis denying and minimizing its load.

“It was a nonsense search, I realized it later, but it was the thing that struck me most... looking for a cause that maybe there wasn’t... I thought the fault was mine, some negligence or something that had brought me to the disease, the 40 years before... what was wrong? Absurd to think but my thought was there...” [6]

“Initially this path was a little bit ‘... as in dealing with all the diseases you always found difficulties…I made like a lineup to understand how I had to go through my life, I told myself ‘I want to face open-mouth or I want to drag myself throughout my life... I told myself I am studying a path that would help me to leave this... this difficulty that I have encountered” [7]

### Experience of the Illness Journey: Disease and Treatment

Patients perceived the disease as invisible, something that does not affect their lives; at the same time, they had difficulties in thinking or talking about death.

In particular, patients reported the difficulty in creating a mental space to think about the disease during their life, which is probably related to the unsolved process of coping with the diagnosis.

“I want to continue, let’s say, I would like to continue hum... always following that instinct that led me, when there are empty spaces in which I’m not doing or thinking about something else, I have a moment of reflection, to occupy thinking about new things and not to what is happening to me, trying to continue my life normally... until now I have succeeded” [6].

Some patients referred in an ambivalent way both to the disease, as something present but not visible, and to the treatment, as the thing that testifies the reality of the illness; at the same time, therapy was something to be grateful for.

“[the disease] is like a daily and silent war because maybe from the outside, all in all, it is not like those who do chemotherapy, it’s an everyday war, twice a day you remember this thing, then, all in all, I learned to live together with it, it becomes an automatic thing like eating, drinking, so in short” [4].

“I am one in a million, a Highlander…” [14].

Thus, patients reported a good adaptation to the treatment, something that began to be integrated in the patient’s life; however, all the individuals referred to the treatment as essential for life and survival.

“Doctors proposed to me to move on to this new drug and…I can say that I lived in a positive way even if at home I was afraid to take this new substance...let’s say yes, it was something dramatic rather than tragic; however, I learned to live with it, just because I saw that there was this drug I did not live it [bad]...it started to be part of me” [4].

“The drug was a perfect cure … it was a life saver” [1]

“We were in the Amazon rainforest and I told a friend ‘this drug is a lifesaver for me, that’s what I need to take!’ I have it with me but I might lose this backpack so... please carry a dose for me...just in case.” [2]

### Experience of TFR Failure

Patients’ perception of the TFR failure, its meaning and its impact can be understood when their individual experience and disease history are considered. In particular, the experience of failure proved to be linked to the imagined or lived experience of stopping treatment. Patients’ experience of failure always reflects a slightly negative emotional experience.

“I did not see it (the trial discontinuation) in such a negative, such a dramatic way that I would say ‘from now on how will I cope with life? How will I do it?’ I accepted it pretty well, I think I accepted it well…because undoubtedly that day that doctors told me about the disease I thought... I said: ‘Why I got sick?”’ [9]

“This course of treatment presented some negativity, some aspects that could create me psychological difficulties... these things here (sexual difficulties)... it was not a path just so negative or so positive... a normal path, let’s say” [6].

Patients referred to the trial discontinuation as “a little bit of…not dejection…but a bitter pill” and as “stumbling” during the care pathway.

“I have lived the path a bit like a bicycle climb... you come halfway up and they tell you that the road is closed... it left me a little bit like this...” [12].

“Undoubtedly you hope... how can I say … you let yourself believe that you manage to suspend the drugs and return to life...actually I have a normal life, I have only to take these pills but my life is in some way normal…we have tried to remove it completely, it did not happen and I went ahead in the same way” [6].

“If I have managed to be included in that group [of patients eligible for TFR] I would have lived it a bit like a prize after so many years of treatment, then, all in all, compared to other situations I tend to say ‘I have to thank for it’...that’s ok after all” [4].

“When I was small, I had a puppet of the type that, when you beat him, always gets back on its feet... I am like this puppet who always puts himself in position... I do not know if it’s thoughtlessness” [2].

The ambivalent view of the clinical trial and of patient’s early discontinuation was also related to the comforting feeling of being engaged and more closely cared for during the trial, so that the discontinuation has been seen as “an abandonment.”

“This [EOP] has a little ‘knocked out because hum... because I felt good, I felt followed and the check-ups were often... and now I see myself as a bit’ set aside. I always continue with the pills and my therapy, but I am no longer part of this protocol, I am a bit ‘thrown down” [11].

The perception of unpleasantness gains meaning when linked to the idea of stopping treatment, which is seen as something useful but at the same time demanding: the therapy often appears to be the only sign that still remind of the presence of the disease, but it also represents a sort of “comfort zone” for patients, something they are used to, and that represented a sort of lifesaver.

*“I suspended the treatment for a week... and now I’ll have to restart with it; however, I... I’m in doctors’ hands because... they are the ones who give me life... if I suspend [the treatment] I know that the disease will reappear within a month” [9]*.

### The Impact of Relapse on the Patient’s Life

CML was perceived as something invisible but present, like a ghost that permits us to conduct normal lives but at the same time undermines future plans, changing the perspective in which patients live their lives; patients reported to live for the day, without too many expectancies or long-term plans.

“I don’t want to have high expectations or hopes, I follow the course of events…I say to myself ‘let’s see if it is something that can lead me towards something good. You will discover it by living” [10].

“Honestly I am living my life day by day... I do not expect to do great things later because I do not know if... if I will get there... I do not have... thoughts about the future... tomorrow is another day... as the disease has a sudden outbreak” [9]

*“They reassured me saying that there are other drugs…let’s say, I don’t live the day without worries about the future, because obviously I think about the future” [4]*.

“The road has changed... for e.g., Italy is now a country where work is not easily found. I am going to look for work somewhere else, gain experience abroad. However, there is always this unknown about going abroad as I always have this factor (disease) to consider” [8].

In particular, the disease seems to have a sort of deadly effect on the procreative plans, and reproductive successes appeared to be “miracles.” Moreover, with regard to their children, patients reported to be worried about not being able to fully perform their parental roles and functions.

“Since the beginning, they clearly told me that I probably would not have children...I remember that in front of the doctor I started crying, because maybe at 27 it was something that I was postponing … I remember that I cried so much…then I started to accept the idea…and that is not something I suffer from, but maybe if it did not happen I would have had (a child) like everyone else” [4].

“After the illness, my wife and I decided not to have another child... all in all it’s a small regret. But that’s ok” [3]

“They treated me... they gave me therapy … and I had a child... that is, I managed to have a child...I thought that the child could have problems and so on, but he was born healthy” [9].

“Sometimes, when I’m alone in my car, I also have these weaknesses... like any human person, let’s say, I don’t rock the world but I’m just starting to think, I’ve got my family... And I worry not about myself but about my children” [6].

## Discussion

This sub-study represents one of the few available studies that face up to the emotional aspects of living the CML in the new era where TKIs offer a long survival with, in the majority of cases, a good QoL. Despite new drugs for CML offer high chances of optimal responses (Hochhaus et al., 2016), our interviews suggest that patients could experience emotional distress during the illness journey.

We suggest that our qualitative approach allows to explore the inner emotional experience in a way that integrates the most common quantitative approach: through a bottom-up method and a “majeutical”, way individuals could exteriorize some introspective aspects adding additional information to the *a priori* items of questionnaires.

In particular, it was interesting that at the beginning of the narration, all patients remembered the moment of diagnosis, even if the interviewer, thus exemplifying that this ghost still survives even after several years of an effective treatment, did not require it. On the other hand, in spite of the efficacy results observed with nilotinib, in all cases the fear of long-term planning remains, thus highlighting a further aspect previously undetected by the literature based on quantitative methods (i.e., questionnaires).

As for the diagnosis, it is true that our patients firstly remembered the moment of the diagnosis as a shock, like a traumatic break, which is difficult to express and that probably is not yet metabolized, but our patients tried to rapidly reorganize their lives around the idea that “CML is curable, and thanks to the treatment they could have the opportunity of conducting normal lives.” This result is consistent with the findings of a recent study exploring, through narrative diaries, both the impact of the disease on CML patients’ everyday lives and the psychological impact of their being presented with the chance to suspend their therapy (Graffigna et al., 2017). The study highlighted how patients described the communication of the diagnosis as a “bolt from the blue,” something that was unexpected and disrupted patients’ lives.

The drama of the diagnosis as lived by patients had some common characteristics with the emotional experience of receiving a piece of bad news. There is a wide literature on bad news highlighting the fact that the doctor’s communication of a bad news is worse (and the processing thereof more difficult and traumatic) as the patients is farther from it (Buckman, 1992). Indeed, CML patients usually did not experience symptoms or problems before the diagnosis and diagnosis often occurs by chance (e.g., regular blood tests or blood donation). Buckman suggested that the physician should left time and space to patients emotional reaction and processing of the bad news during its communication (e.g., step-by-step communication).

Moreover, CML patients reorganized their lives after the diagnosis, and this process seemed to be driven by the dramatic change of the CML panorama: the disease has shifted from a mortal to a chronic condition, and nowadays the perspective is that it could become curable. This rapid progress seemed hard to integrate in patients’ mind, and often did not settle in a congruent reorganization of the patients’ perspective of disease. Some patients described their CML experience as a “silent war”: this metaphor was often retrievable in cancer patients’ narratives (Skott, 2002), and was found to be particularly powerful in revealing CML patients’ illness experience (Graffigna et al., 2017). Indeed, it highlights the ambivalence of the emotional and cognitive experience of being a CML patient that originates from the moment of the diagnosis, like a wound beyond healing. Our data interpretation seems to be coherent with the whole illness journey, particularly the ambivalent connection to the treatment and its discontinuation.

Another innovative aspect of our study is that to our knowledge it is the second study facing the TFR in CML from the psychological point of view. The Australian group (Villemagne Sanchez et al., 2018) recently reported the answers from 84 patients on their feeling about the TFR: half of them received imatinib, and the rest nilotinib or dasatinib. No significant difference in the emotional perception of TFR according to the type of TKI was observed. Confidence in the physician and the possibility of eliminating toxicities resulted in the most frequently reported reasons for willingness to attempt TFR. In that series, only 7 participants expressed anxiety or fear about stopping treatment (Villemagne Sanchez et al., 2018).

Moreover, the study by Graffigna et al. (2017), exploring reactions to the possibility of interrupting treatment, is in some way a pre-step of our study that interviewed only patients who really entered into a clinical trial with the goal of TFR.

Our findings showed that the way in which patients gave sense to the experience of TFR depended on their whole journey, beginning from the diagnosis. TFR was also lived in an ambivalent way: with hope, as a possibility to discontinue therapy, but also with fears, because often patients linked the treatment with their life safety. In this regard, many patients who discontinue the TKI (estimated to be up to 30%) experience musco-skeletal pain (Hochhaus et al., 2017), which is described as “TKI withdrawal syndrome” (Richter et al., 2014; Galimberti et al., 2015). This possible event also needs to be very carefully explained to the patients, as the first thought when experiencing those symptoms might be that they have a relapse.

Our interviews showed that patients were reluctant to think that the success of TFR could lead to healing. Indeed, we have to consider that for many years physicians told patients that TKI was saving their lives, and that they needed to take it every day because compliance is the first step for achieving a good response (and survival). Now the physicians need to deal with these new messages, as newest clinical data clearly show that a life without drugs and a potential “operative cure” is possible. Understanding the patient’s perspective and lived experiences is undoubtedly an asset for more efficient health interventions and improved clinical compliance.

Finally, the way in which our sub-study CML patients gave sense to the experience of the early trial discontinuation in the light of their illness history is likewise a relevant finding of our work: this result is really new, but some studies seemed to point in the same direction, as they proved that patients’ emotional experience of being involved in a clinical trial depends on the feelings related to the disease itself and experienced before entering into a trial. Wineman et al. (1996), for example, suggested that subjects entering into a clinical trial for multiple sclerosis with high levels of illness uncertainty and stress were likely to experience mood disturbances and felt less hopeful about treatment effectiveness. Moreover, the pattern of relationships among uncertainty, coping, hopefulness, and mood did not change throughout participation in the experimental trial (Wineman et al., 2003).

### Limits

The methodology used, although fundamental for the aim, implies some limits that the qualitative research could present. In particular, the study is based on a small sample of CML patients. However, we interviewed only patients receiving nilotinib as part of the ENESTPath trial; this ensures a population that comes from various hospitals in different areas of Italy and that is homogenous in terms of treatment duration and therapy management. As in all qualitative studies, the findings are not statistically generalizable, but offer some clinically useful insights to improve CML patients’ care. The study constitutes an interesting stimulus to favor a process of understanding CML from a patient-centered view, which could help physicians to improve their relationship with patients and the confidence that emerged as fundamental in order to stimulate in patients the willingness to being engaged in a clinical trial.

## Conclusion

The Italian sub-study clearly showed how CML patients perceive early trial discontinuation, and the way they give sense to it appears to have a strict relationship with individual disease experience. This qualitative analysis helps in understanding patients’ perspective, both in terms of getting access to the inner subjective experience of having CML and of being involved in a trial or its cessation. The insights provided show once again that patients’ experience of the diagnosis, in its primordial ambivalent nature, with the co-existence of opposite feelings, influenced patients’ perception of the disease, of the treatment, as well as of the eventual patient’s failure of the clinical trial. There is a need to develop strategies to cope with patients’ emotional experience and guarantee that their psycho-emotional wellbeing is one of the objectives of each new clinical trial.

## Author Contributions

All authors contributed to conception and design of the work and interpretation of data, and revised the manuscript critically for important intellectual contents and gave their final approval of the version to be published. LB, SG, CB, MB, EC, AC, FF, AI, FL, CM, EMO, GR-C, and SiS contributed to the acquisition of data. LB, SG, and EV analyzed the data. LB and EV drafted the manuscript.

## Conflict of Interest Statement

LB received a research fellowship from her Institution on the project titled “CML patient’s voice: A pilot study exploring the emotional experience of patients during CAMN107AIC05 study and its discontinuation” funded by Novartis Pharma AG. She declares it does not influence her role and contribution to the manuscript. SG, CB, MB, EC, AC, FF, AI, FL, CM, EO, GR-C, and SiS received economical support for some studies from Novartis, but it did not influence their roles and their contribution to the manuscript. ShS and JH are employees of Novartis Pharma AG. EV received grant support, paid to her institution, from Novartis for the research project titled “CML patient’s voice: A pilot study exploring the emotional experience of patients during CAMN107AIC05 study and its discontinuation.” She declares it does not influence her role and contribution to the manuscript. This sub-study was funded by Novartis Pharma AG. The study participants have not received funds for their participation in the study. The results presented in this manuscript have not been previously published except an abstract, including preliminary data, that was presented but not selected for presentation at the 59th Annual Meeting and Exposition (December 9–12, 2017); however, the abstract was published online-only on the ASH abstracts site on December 8, 2017, and became part of the permanent ASH and Blood abstracts archive.
